# Does Anterior Oblique Sling Training Reduce Groin Pain and Enhance Hip Mobility, Adductor Strength, and Performance in Soccer Players with Groin Strain?

**DOI:** 10.5812/aapm-162623

**Published:** 2025-07-19

**Authors:** Fatemeh Ahmadi, Hooman Minoonejad, Seyed Hamed Mousavi, Arash Khaledi

**Affiliations:** 1 Department of Sports Injury and Biomechanics, Faculty of Sport Sciences and Health, University of Tehran, Tehran, Iran; 2 Department of Physical Education and Sport Sciences, Ma.C., Islamic Azad University, Mashhad, Iran

**Keywords:** Groin Strain, Anterior Oblique Sling, Rehabilitation, Hip Mobility, Adductor Strength, Soccer

## Abstract

**Background:**

Groin strains are common in soccer and often lead to prolonged recovery and high recurrence. Traditional rehabilitation may overlook the role of integrated trunk-limb coordination, which is essential in multidirectional sports. The anterior oblique sling (AOS) system, involving the obliques, adductors, and abdominal fascia, is key in dynamic stability and force transfer.

**Objectives:**

Evaluate the effects of AOS training on pain, hip mobility, adductor strength, and performance in soccer players with groin strain.

**Methods:**

In this quasi-experimental study, 30 male semi-professional footballers (18 - 30 years, with ≥ 5 years' experience and confirmed groin strain) were allocated to either an 8-week supervised AOS training program (3x/week, 40 - 60 min/session; n = 15) or a control group (n = 15). Outcomes assessed pre- and post-intervention included pain [Visual Analog Scale (VAS)], hip mobility, isometric adductor strength (dynamometer), and change-of-direction and acceleration test (CODAT). The data were analyzed via repeated-measures ANOVA and nonparametric tests.

**Results:**

The AOS group experienced a 35.6% reduction in pain (VAS: 4.66 ± 0.61 to 3.00 ± 0.92; P = 0.001), unlike the control group. Hip mobility significantly improved in the AOS group (abduction: +4.47°, flexion: +8.87°, extension: +2.54°, internal rotation: +5.73°, external rotation: +3.93°; all P ≤ 0.04, η^2^ = 0.14 - 0.36), with no similar gains in the control group. Adductor strength increased by 19.8% in the AOS group (P = 0.001, η^2^ = 0.32) compared to 4.6% in the control group (P = 0.17). Performance improved by 8.0% in the AOS group (CODAT time: 7.03 ± 0.46 to 6.47 ± 0.41 s; P = 0.001, η^2^ = 0.51), with no change in the control group.

**Conclusions:**

The AOS training effectively reduces groin pain and enhances functional outcomes in soccer players. It offers a promising, chain-based rehabilitation approach for dynamic sports.

## 1. Background

Groin strain injuries represent a prevalent and functionally limiting condition in soccer, accounting for a significant proportion of time lost from training and competition (1). These injuries primarily affect the hip adductor complex, notably the adductor longus, magnus, and brevis, resulting in persistent pain, reduced hip mobility, diminished adductor muscle strength, and impaired physical performance (2). Soccer is a globally popular sport, engaging approximately 265 million players worldwide, including 25 million registered with the United States Soccer Federation alone (3, 4). The high-intensity, multidirectional demands of the game, such as sprinting, cutting, and kicking, place substantial mechanical stress on the adductors and surrounding structures, increasing the risk of strains and overuse injuries (1). Epidemiological data highlight a greater susceptibility to groin injuries among male soccer players: 59% Report at least one episode (compared to 45% of females), with an average weekly prevalence of 29% (range: 23% - 32%), an incidence rate of 1.0 per 1000 hours, and a 3.1 times higher risk than elite female players (1). For example, U.S. collegiate players commonly sustain adductor strains (46.5%) (2), and a study of 17 clubs showed that 21% of players per club experience groin injuries each season, with an incidence of 1.0 per 1000 hours (95% CI 0.9 to 1.1) and a median absence of 10 days per injury (5).

Despite the potential for underreporting, the frequency of groin strains and their impact on athletic capacity due to factors such as muscle imbalance and eccentric contraction underscore the need for targeted research, especially in semi-professional settings (4). Traditional management strategies have often focused on rest, analgesia, and isolated muscle strengthening (6). However, recent attention has shifted toward kinetic chain-based interventions that better reflect the functional demands of the sport (7). One such method is anterior oblique sling (AOS) training, grounded in the neuromuscular synergy theories of Vladimir Janda (8) and the myofascial chain concepts proposed by Myers (9). The AOS, comprising the external obliques, contralateral internal obliques, adductors, and abdominal fascia, plays a pivotal role in trunk stabilization and the transfer of force between the upper and lower extremities, particularly during rotational and multidirectional tasks. Dysfunction within this sling can disrupt core stability and neuromuscular control, leading to increased strain on the groin and pelvis (8, 9). The AOS training aims to restore coordinated function across this chain, enhancing pelvic alignment, trunk control, and dynamic lower-limb stability. This integrative approach addresses not only local symptoms such as pain but also upstream and downstream contributors to impaired movement (9), thereby aligning rehabilitation with the actual biomechanical demands of soccer. Despite its theoretical promise, to our knowledge, no empirical research has specifically evaluated the effects of AOS training on soccer players with groin strains.

## 2. Objectives

This study aimed to evaluate the effects of an 8-week AOS training program on groin pain (primary outcome), as well as hip mobility, adductor strength, and functional performance (secondary outcomes) in soccer players with groin strain. We hypothesized that targeting neuromuscular deficits throughout the kinetic chain would contribute to more effective rehabilitation and improved performance in soccer players with groin strain.

## 3. Methods

### 3.1. Study Design and Participants

This quasi-experimental pretest-posttest study included 30 male semi-professional footballers (aged 18 - 30) with groin injuries (3 - 6 months duration), recruited from sports clinics in Tehran. Based on power analysis (G*Power v3.1.9.7; α = 0.05, power = 0.80), they were equally assigned to exercise (n = 15) or control (n = 15) groups using matched allocation (age and experience), as randomization was not feasible. The study was approved by the University of Tehran Ethics Committee (IR.UT.SPORT.REC.1402.020) and followed the Declaration of Helsinki.

### 3.2. Inclusion Criteria

Eligibility required: (1) male footballers aged 18 - 30; (2) ≥ 5 years of semi-professional experience; (3) groin strain diagnosed clinically within 3 - 6 months; (4) Body Mass Index (BMI) 18.5 - 24.9 kg/m^2^; (5) no ongoing groin rehabilitation. Participants provided informed consent and had no confounding musculoskeletal or neurological conditions.

### 3.3. Exclusion Criteria

Participants were excluded for: (1) Severe groin pain limiting safe participation; (2) missing more than 2 consecutive or 3 non-consecutive sessions in 8 weeks; (3) unwillingness or inability to complete post-testing; or (4) hip or groin surgery within the past year. External physical therapy during the study also led to exclusion.

### 3.4. Outcome Measures

#### 3.4.1. Pain

Pain intensity was assessed using a 100-mm Visual Analog Scale (VAS), ranging from 0 (“no pain”) to 10 (“worst imaginable pain”). The VAS has shown excellent reliability (inter-rater ICC = 1.00; test-retest ICC = 0.99) in prior studies (10).

#### 3.4.2. Hip Range of Motion (ROM)

##### 3.4.2.1. Abduction

**Figure 1.**
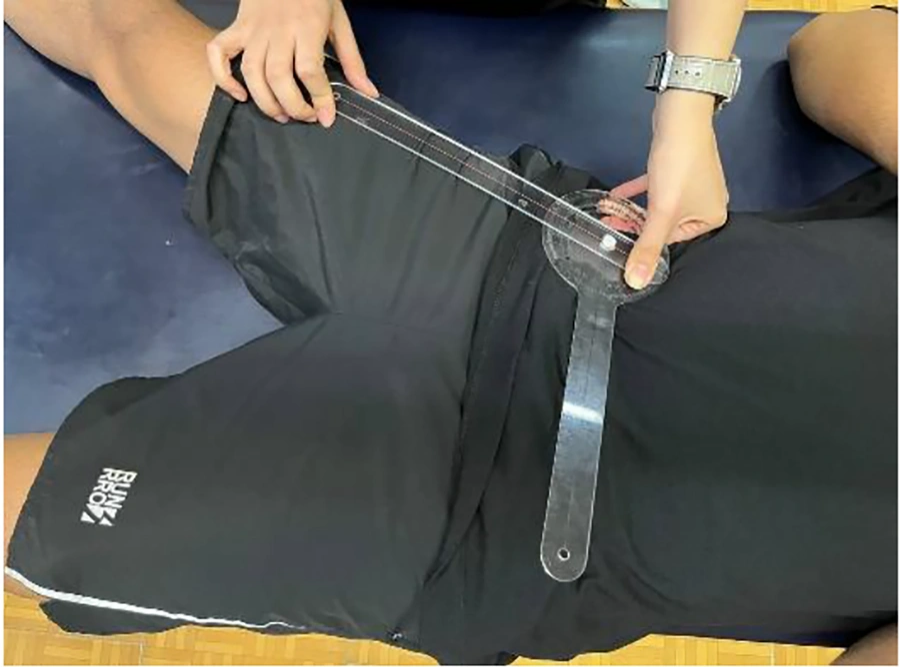
Goniometric measurement of hip abduction in supine position, aligned with anatomical landmarks

With the participant supine, pelvis neutral, and limbs aligned, the examiner stabilized the pelvis and guided the femur. The participant actively abducted the hip. A goniometer measured the angle (fulcrum on ASIS, stationary arm across ASISs, moving arm along the femur toward the patella). Starting position: 90° (Figure 1). 

##### 3.4.2.2. Flexion

**Figure 2.**
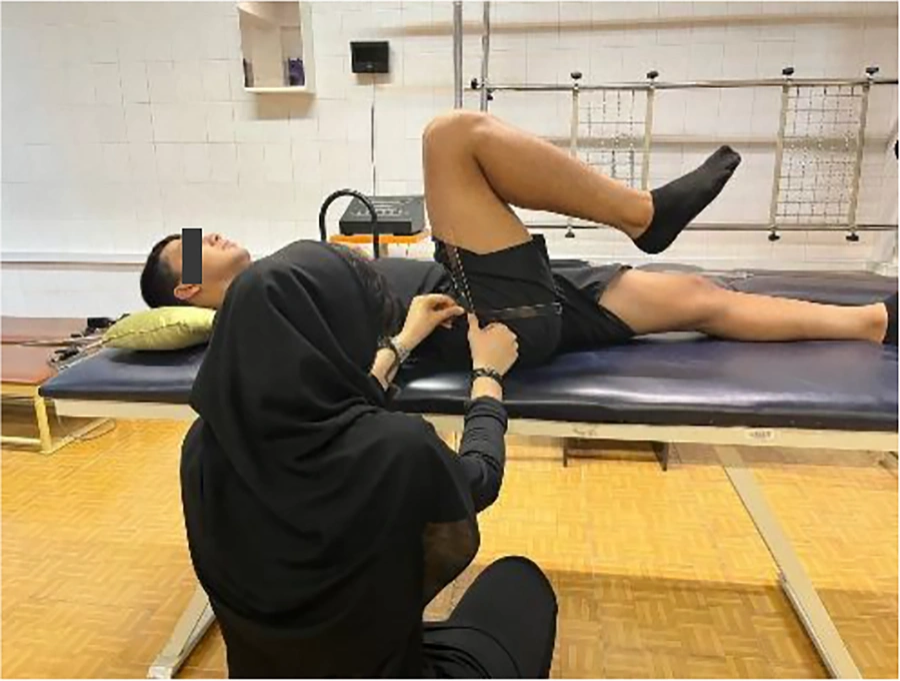
Hip flexion measurement: Supine participant with goniometer positioned at greater trochanter, stationary arm on pelvis, and moving arm along femur.

In a supine position with the non-tested leg neutral, the participant actively flexed the tested hip with a bent knee. A goniometer was used (fulcrum on the greater trochanter, stationary arm along the mid-axillary line, moving arm aligned with the femur toward the lateral epicondyle; Figure 2). 

##### 3.4.2.3. Extension

**Figure 3.**
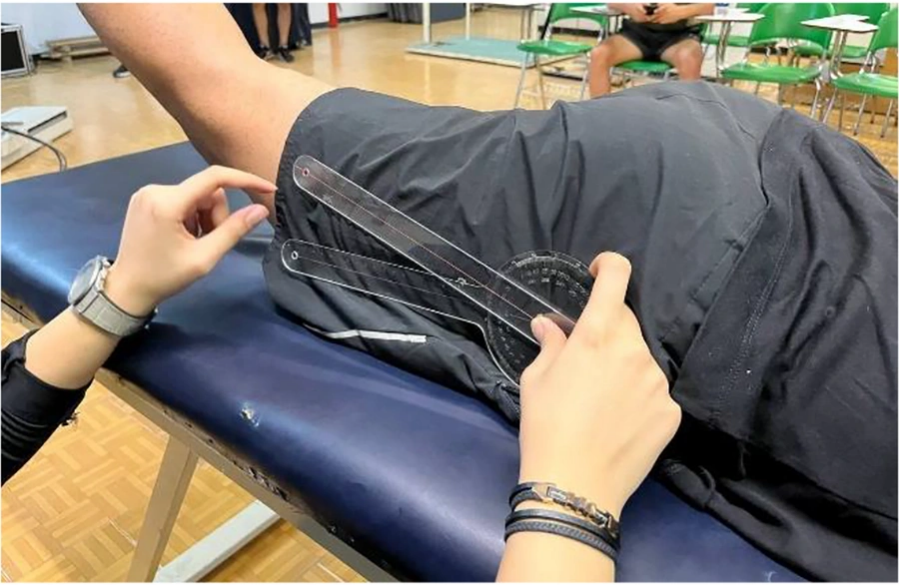
Prone hip extension measurement: Goniometer placed at greater trochanter, measuring angle between trunk and femur.

In a prone position with hips and knees neutral, the participant actively extended the tested hip while the examiner stabilized the pelvis. ROM was measured with a goniometer (fulcrum on the greater trochanter, stationary arm along the mid-axillary line, moving arm aligned with the femur’s lateral midline; Figure 3). 

##### 3.4.2.4. Internal/External Rotation

**Figure 4.**
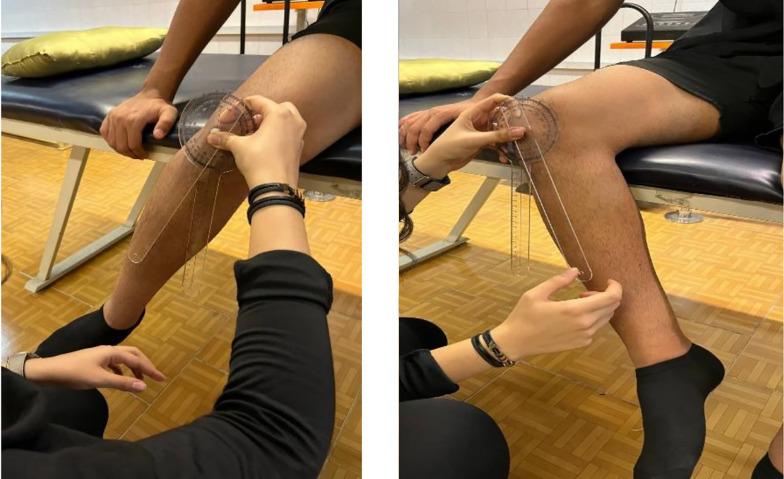
Seated hip rotation measurement (internal left, external right) with 90° hip/knee flexion

Seated at the table edge with hips and knees at 90° flexion, participants actively rotated their hips. A goniometer measured ROM (fulcrum on the patella, stationary arm perpendicular to the floor, moving arm aligned with the tibial crest; Figure 4). 

Goniometers show excellent reliability (ICC > 0.90) and acceptable validity for hip ROM, despite slight overestimation (11).

### 3.5. Isometric Strength of Hip Adductors

**Figure 5.**
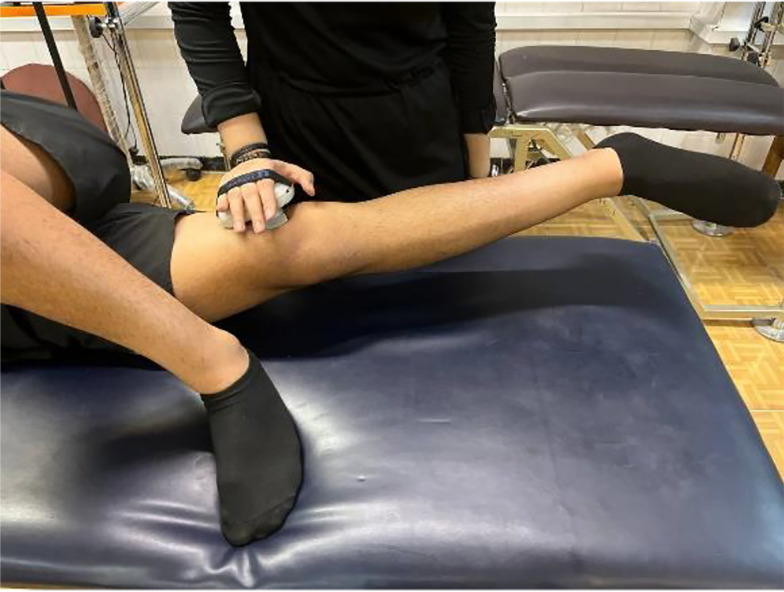
Dynamometric assessment of isometric hip adductor strength in side-lying position, with device positioned above medial femoral epicondyle

In a side-lying position (tested hip extended, opposite leg flexed at 90°), participants performed maximal isometric adduction against a dynamometer placed 5 cm above the medial femoral epicondyle (3 × 5 sec trials, 15-sec rest). The mean force (kg) was recorded (Figure 5). This method is highly reliable (intra-rater ICC = 0.70 - 0.89; inter-rater ICC = 0.66 - 0.87) and valid (ICC ≥ 0.90) for athletes (12, 13).

### 3.6. Functional Performance

**Figure 6.**
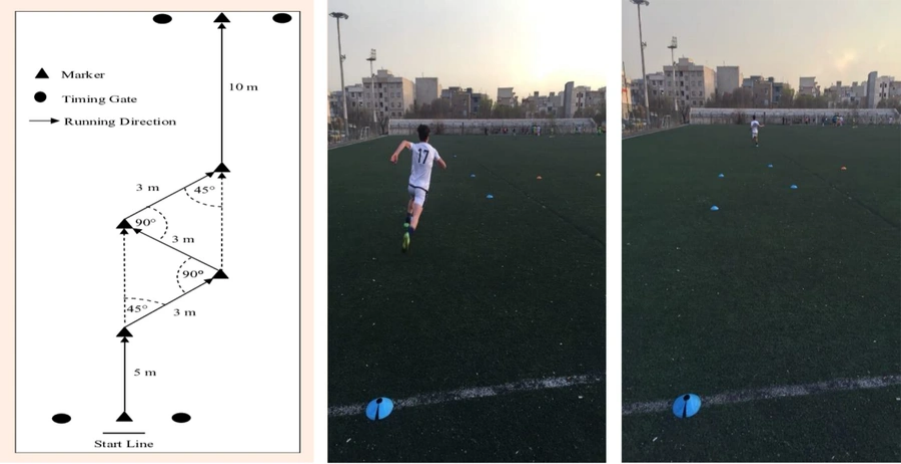
Change-of-direction and acceleration test (CODAT) course schematic (right) with execution photos: Start (center) and finish (left).

The change-of-direction and acceleration test (CODAT) evaluated football players' agility through a 24-meter course comprising an initial 5-meter sprint, three 3-meter sprints with 45° and 90° directional changes, and a final 10-meter sprint, with four cones marking turns and standardized timing equipment. The CODAT shows strong reliability (ICC = 0.84, CV = 3.0%) and validity with Illinois agility (R = 0.92) and 20m sprints (R = 0.75 - 0.76), effectively assessing change-of-direction ability (14) (Figure 6). 

### 3.7. Interventions

This 8-week program included three weekly 40 - 60 minute sessions, each with a 5 - 7 minute warm-up, 40 - 50 minutes of main exercises, and a 3 - 5 minute cool-down, following FITT principles (15). The regimen incorporated 11 targeted exercises: (1) Active isolated groin stretching with a TheraBand, (2) dynamic and static adductor stretches, (3) weighted side-lying hip adduction, (4) supine TheraBand adduction at three angles, (5) eccentric adductor slides, (6) resistance band slides, (7) AOS exercises in multiple positions, (8) balance training, (9) medicine ball throws, (10) half-kneeling rotational throws, and (11) TheraBand AOS combined with chest presses (see Appendix 1 in Supplementary File). In contrast, the control group maintained their standard 15-minute warm-up routine (16).

### 3.8. Statistical Analyses

Descriptive statistics summarized sample characteristics. The Shapiro-Wilk test assessed data normality. A repeated measures ANOVA evaluated time (pre vs. post) × group (exercise vs. control) effects. Analyses were conducted using SPSS v26, with significance set at P < 0.05.

## 4. Results

Table 1 presents participant demographics, showing no significant baseline differences between groups in age, height, weight, BMI, or sport experience (P > 0.05), indicating group homogeneity. Table 2 summarizes intervention effects: The AOS group showed significant improvements in pain, hip ROM, adductor strength, and performance, whereas the control group had no notable changes.

**Table 1.** Demographic Characteristics and Homogeneity of Participants ^a^

Variables	Control Group (N = 15)	Experimental Group (N = 15)	P-Value
**Age (y)**	22.73 ± 3.73	23.66 ± 3.39	0.48
**Height (m)**	1.76 ± 0.05	1.76 ± 0.06	0.14
**Weight (kg)**	68.93 ± 5.35	66.86 ± 5.69	0.31
**BMI (kg/m** ^**2**^ **)**	22.03 ± 1.66	22.12 ± 1.44	0.86

Abbreviations: SD, standard deviation; BMI, Body Mass Index; m, meters; kg, kilograms.

^a^ Values are expressed as mean ± SD.

**Table 2.** Summary of All Outcome Measures Pre- and Post-intervention (Mean ± SD)

Variables	Pre-test	Post-test	P (Within)	P (Between)	Effect Size (η^2^)
Pain (VAS)					
Experimental	4.66 ± 0.61	3.00 ± 0.92	0.001	0.001	-
Control	4.73 ± 0.59	4.53 ± 0.63	0.25	-	-
**Hip abduction ROM (°)**					
Experimental	44.66 ± 1.21	49.13 ± 1.22	0.001	0.01	0.14
Control	43.73 ± 1.50	45.26 ± 1.53	0.34	-	-
**Hip flexion ROM (°)**					
Experimental	90.13 ± 9.03	99.00 ± 3.92	0.001	0.03	0.24
Control	92.73 ± 7.77	94.06 ± 7.74	0.46	-	-
**Hip extension ROM (°)**					
Experimental	22.66 ± 3.84	25.20 ± 3.93	0.001	0.04	0.36
Control	21.93 ± 0.03	22.53 ± 3.11	0.09	-	-
**Hip internal rotation ROM (°)**					
Experimental	42.60 ± 5.23	48.33 ± 5.10	0.001	0.01	0.34
Control	42.06 ± 5.67	43.40 ± 5.61	0.11	-	-
**Hip external rotation ROM (°)**					
Experimental	44.00 ± 6.17	47.93 ± 5.37	0.001	0.01	0.21
Control	42.53 ± 4.50	43.33 ± 4.32	0.32	-	-
**Adductor strength (kg/BW)**					
Experimental	15.17 ± 4.62	18.17 ± 4.07	0.001	0.008	0.32
Control	13.84 ± 3.02	14.48 ± 2.82	0.17	-	-
**Physical performance (sec)**					
Experimental	7.03 ± 0.46	6.47 ± 0.41	0.001	0.03	0.51
Control	6.90 ± 0.57	6.88 ± 0.58	0.82	-	-

Abbreviations: SD, standard deviation; ROM, range of motion; VAS, Visual Analog Scale; BW, body weight; sec, seconds; η^2^, Eta squared; P (within), within-group significance; P (between), between-group significance.

### 4.1. Pain

The experimental group showed significant pain reduction (4.66 ± 0.61 to 3.00 ± 0.92; P = 0.001), while the control group showed no change (4.73 ± 0.59 to 4.53 ± 0.63; P = 0.001). A Mann-Whitney U test confirmed significant between-group differences (P = 0.001). Non-parametric tests were used due to non-normal distribution.

### 4.2. Hip Abduction ROM

The experimental group significantly improved (44.66 ± 1.21° to 49.13 ± 1.22°; P = 0.001), unlike controls (43.73 ± 1.50° to 45.26 ± 1.53°; P = 0.34). Repeated measures ANOVA revealed significant between-group differences (P = 0.01, η^2^ = 0.14), indicating small-to-moderate intervention effects.

### 4.3. Hip Flexion ROM

The experimental group showed significant improvement (90.13 ± 9.03° to 99.00 ± 3.92°; P = 0.001), while the control group did not exhibit a statistically significant change (Pre: 92.73 ± 7.77°; Post: 94.06 ± 7.74°; P = 0.46). Post-intervention between-group differences were significant (P = 0.03, η^2^ = 0.24), demonstrating moderate training effects.

### 4.4. Hip Extension ROM

The experimental group experienced significant improvement (22.66 ± 3.84° to 25.20 ± 3.93°; P = 0.001). In contrast, the control group showed a non-significant change (21.93 ± 0.03° to 22.53 ± 3.11°; P = 0.09). Between-group differences were significant (P = 0.04, η^2^ = 0.36), reflecting large intervention effects.

### 4.5. Hip Internal Rotation ROM

Significant improvement was observed in the experimental group (42.60 ± 5.23° to 48.33 ± 5.10°; P = 0.001). The control group did not show significant change (42.06 ± 5.67° to 43.40 ± 5.61°; P = 0.11). Between-group differences were significant (P = 0.01, η^2^ = 0.34), indicating large intervention effects.

### 4.6. Hip External Rotation ROM

The experimental group demonstrated a significant improvement (44.00 ± 6.17° to 47.93 ± 5.37°; P = 0.001), while the control group did not (42.53 ± 4.50° to 43.33 ± 4.32°; P = 0.32). The between-group difference was significant (P = 0.01, η^2^ = 0.21), showing moderate treatment effects.

### 4.7. Adductor Muscle Strength (kg/body weight)

A significant increase in adductor strength was seen in the experimental group (Pre: 15.17 ± 4.62 kg/BW; Post: 18.17 ± 4.07 kg/BW; P = 0.001). The control group did not experience a significant change (Pre: 13.84 ± 3.02 kg/BW; Post: 14.48 ± 2.82 kg/BW; P = 0.17). Between-group differences were significant (P = 0.008, η^2^ = 0.32), demonstrating large treatment effects.

### 4.8. Physical Performance (Seconds)

The experimental group showed significant improvement in physical performance time (Pre: 7.03 ± 0.46 s; Post: 6.47 ± 0.41 s; P = 0.001). The control group did not exhibit a significant change (Pre: 6.90 ± 0.57 s; Post: 6.88 ± 0.58 s; P = 0.82). Between-group differences were significant (P = 0.03, η^2^ = 0.51), indicating large intervention effects.

## 5. Discussion

This study examined the impact of an 8-week AOS training program on groin pain, hip mobility (flexion, extension, abduction, and adduction), adductor strength, and performance in semi-professional soccer players with groin strain. Results showed that AOS-based rehabilitation led to significant improvements in all areas compared to traditional adductor-focused methods, suggesting that targeting the anterior oblique kinetic chain may be more effective. Athletic groin pain is a multifactorial syndrome, often caused by force transmission imbalances, neuromuscular deficits, and poor lumbopelvic-hip coordination (17). Studies by Harøy et al. (18) and Hölmich et al. (19) confirm that adductor strengthening (e.g., Copenhagen exercise) aids prevention and rehabilitation, but these uniaxial, isolation-focused protocols overlook broader stabilizing systems like the deep core and rotational slings, which are essential for sport-specific multiplanar movements.

For instance, Serner et al. found that while Copenhagen adduction and elastic-resisted hip adduction strongly activate the adductor longus (> 100% nEMG), they minimally engage core and gluteal muscles (20). This lack of proximal stabilizer recruitment reveals a key limitation in traditional rehabilitation: Isolated strength training may not restore integrated lumbopelvic function. Since sports like soccer require rotational control, dynamic stability, and pelvic load transfer (21), this gap between local strength and global coordination calls for more comprehensive interventions (22). Unlike traditional methods (16), our AOS protocol replicated sport-specific multiplanar demands through functional movements like diagonal chops and rotational lunges, synchronously engaging the ipsilateral internal oblique, contralateral external oblique, and adductors (8). This approach aligns with Vleeming et al.'s force closure model, where pelvic stability stems from coordinated myofascial sling activation rather than isolated strength (23). The resulting gains in agility, speed, and pain reduction highlight how restoring natural kinetic chain integration, not just local muscle strength, drives optimal rehabilitation outcomes. This approach reflects modern rehabilitation principles emphasizing motor control and kinetic chain restoration for chronic musculoskeletal issues, as supported by Harris-Hayes et al. regarding hip-related groin pain (24). Our study advances this concept by showing that sport-specific, load-bearing control exercises optimize neuromuscular recruitment and movement patterns. This key finding indicates effective rehabilitation requires not just isolated muscle recovery, but rebuilding integrated sensorimotor systems essential for athletic function (8).

Our results both align and contrast with Chaari et al.'s core training study (21). While their 12-week program improved HAGOS scores without agility gains, our approach enhanced both functional performance and subjective measures. This key difference underscores that isolated core work, though beneficial for pain and segmental control, may not fully restore the sport-specific neuromuscular patterns needed for explosive, high-velocity movements. Current research reveals limitations in traditional groin rehabilitation protocols. Alsirhani et al. showed the Copenhagen exercise restored adductor strength but neglected key return-to-play measures like ROM and function (22). Similarly, Cotellessa et al. achieved only modest gains with their multimodal approach, possibly due to a lack of kinetic chain integration and EMG-guided progression (25). These gaps highlight a critical issue: Focusing solely on isolated strength without addressing neuromuscular integration may lead to incomplete recovery.

Our study supports the value of integrated groin rehabilitation. Unlike traditional compartmentalized methods (16), our protocol combined strength, mobility, and motor control through functional, multiplanar movements. Improvements in both performance and pain likely reflect tissue healing and neuroplastic adaptations, consistent with evidence linking chronic groin pain to altered cortical excitability and proprioceptive deficits (15).

These findings should be interpreted cautiously due to several limitations: A small, homogenous male-only sample, single-center non-randomized design, lack of blinding, and absence of neuromuscular assessments, all of which limit generalizability and mechanistic insight. The 8-week follow-up also precluded evaluation of long-term outcomes such as reinjury risk and sustained improvements. Future studies should include biomechanical and neurophysiological measures, use multicenter RCTs with diverse populations, and explore integrated rehabilitation approaches combining kinetic chain and neurocognitive training.

### 5.1. Clinical Implications

AOS training enhances rehabilitation outcomes by restoring neuromuscular control and kinetic chain function. Its sport-specific design facilitates clinical and on-field application, potentially reducing reinjury risk and improving return-to-play readiness.

### 5.2. Conclusions

An 8-week AOS training program significantly reduced groin pain and enhanced hip ROM (flexion, extension, abduction, and adduction), adductor strength, and functional performance in semi-professional soccer players with groin strain. These findings support the integration of kinetic chain-based rehabilitation targeting the AOS as an effective strategy for both injury recovery and performance optimization in multidirectional sports.

aapm-15-4-162623-s001.pdf

## Data Availability

The datasets generated and analyzed during the current study are available from the corresponding author upon reasonable request. Due to ethical considerations and the need to protect participant confidentiality, the data are not publicly accessible.
