# Supplementary material for: Does Anterior Oblique Sling Training Reduce Groin Pain and Enhance Hip Mobility, Adductor Strength, and Performance in Soccer Players with Groin Strain?
Source: Anesth Pain Med. 2025 Jul 19;15(4):e162623. doi: 10.5812/aapm-162623 (PMC12523628; doi:10.5812/aapm-162623)
Supplement: aapm-15-4-162623-s001.pdf [file aapm-15-4-162623-s001.pdf]

Appendix 1

Exercise	Week 1	Week 2	Week 3
1. Groin stretch with TheraBand (active isolated stretch)	3×10 sec<br>45s rest between sets<br>60s final rest	3×12 sec<br>45s<br>60s	3×14 sec<br>45s<br>60s
2. Adductor muscle stretch (dynamic)	3×10 reps<br>45s<br>60s	3×12<br>45s<br>60s	3×14<br>45s<br>60s
3. Adductor muscle stretch (static)	3×10 sec<br>45s<br>60s	3×12<br>45s<br>60s	3×14<br>45s<br>60s
4. Side-lying hip adduction with ankle weight	3×10 reps<br>45s<br>60s	3×12<br>45s<br>60s	3×14<br>45s<br>60s
5. Hip adduction at three angles with the TheraBand	3×10 reps<br>45s<br>60s	3×12<br>45s<br>60s	3×14<br>45s<br>60s
6. Eccentric adduction using Val slide	3×10 reps<br>45s<br>60s	3×12<br>45s<br>60s	3×14<br>45s<br>60s
7. Dynamic strength adduction exercise with the TheraBand	3×10 reps<br>45s<br>60s	3×12<br>45s<br>60s	3×14<br>45s<br>60s
8. Anterior oblique sling training with TheraBand (Level 1: Supine)	3×10 reps<br>45s<br>60s	3×12<br>45s<br>60s	3×14<br>45s<br>60s
9. Level 2: Kneeling on both knees	-	3×10 sec<br>45s<br>60s	3×12<br>45s<br>60s
10. Level 3: One knee down, other knee at 90°, foot on ground	-	-	3×10 sec<br>45s<br>60s

Week 4–6

Exercise	Week 4	Week 5	Week 6
1. Groin stretch with TheraBand	3×14 sec<br>45s<br>60s	4×12 sec<br>45s<br>60s	4×14 sec<br>45s<br>60s
2. Adductor stretch (dynamic)	3×14 reps<br>45s<br>60s	4×12<br>45s<br>60s	4×14<br>45s<br>60s
3. Adductor stretch (static)	3×14 sec<br>45s<br>60s	4×12<br>45s<br>60s	4×14<br>45s<br>60s
4. Side-lying hip adduction with/ ankle weight	3×14 reps<br>45s<br>60s	4×12<br>45s<br>60s	4×14<br>45s<br>60s
5. Hip adduction in 3 angles w/ TheraBand	3×14 reps<br>45s<br>60s	4×12<br>45s<br>60s	4×14<br>45s<br>60s
6. Eccentric adduction with Val slide	3×14 reps<br>45s<br>60s	4×12<br>45s<br>60s	4×14<br>45s<br>60s
7. Dynamic strength adduction with the TheraBand	3×14 reps<br>45s<br>60s	4×12<br>45s<br>60s	4×14<br>45s<br>60s
8. Ant. oblique sling with TheraBand (Level 1: Supine)	3×14 reps<br>45s<br>60s	4×12<br>45s<br>60s	4×14<br>45s<br>60s
9. Level 2: Kneeling on both knees	3×14 sec<br>45s<br>60s	4×12<br>45s<br>60s	4×14<br>45s<br>60s
10. Level 3: One-knee, other 90°	3×12 sec<br>45s<br>60s	3×14<br>45s<br>60s	4×12<br>45s<br>60s
11. Level 4: Lunge position	3×10 reps<br>45s<br>60s	3×12<br>45s<br>60s	3×14<br>45s<br>60s
12. Kneeling on balance disc (stability)	3×10 sec<br>45s<br>60s	3×12<br>45s<br>60s	3×14<br>45s<br>60s
13. "Woodchopper" with medicine ball, standing	-	3×10 reps<br>45s<br>60s	3×12<br>45s<br>60s
14. Rotational throw to wall, kneeling (1 knee, 90°)	-	-	3×10 reps<br>45s<br>60s

Week 7–8

Exercise	Week 7	Week 8
1. Groin stretch with TheraBand	4×14 sec<br>45s<br>60s	4×14 sec<br>45s<br>60s
2. Adductor stretch (dynamic)	4×14 reps<br>45s<br>60s	4×14 reps<br>45s<br>60s
3. Adductor stretch (static)	4×14 sec<br>45s<br>60s	4×14 sec<br>45s<br>60s
4. Side-lying hip adduction with weight	4×14 reps<br>45s<br>60s	4×14 reps<br>45s<br>60s
5. Adduction at 3 angles with TheraBand	4×14 reps<br>45s<br>60s	4×14 reps<br>45s<br>60s
6. Eccentric adduction with Val slide	4×14 reps<br>45s<br>60s	4×14 reps<br>45s<br>60s
7. Dynamic adduction with TheraBand	4×14 reps<br>45s<br>60s	4×14 reps<br>45s<br>60s
8. Ant. oblique sling with TheraBand (Level 1: Supine)	4×14 reps<br>45s<br>60s	4×14 reps<br>45s<br>60s
9. Level 2: Kneeling on both knees	4×14 sec<br>45s<br>60s	4×14 sec<br>45s<br>60s
10. Level 3: One-knee, other 90°	4×14 sec<br>45s<br>60s	4×14 sec<br>45s<br>60s
11. Level 4: Lunge position	4×12 reps<br>45s<br>60s	4×14 reps<br>45s<br>60s
12. Kneeling on disc, balance	4×12 sec<br>45s<br>60s	4×14 sec<br>45s<br>60s
13. "Woodchopper" with medicine ball	3×14 reps<br>45s<br>60s	4×12 reps<br>45s<br>60s

Exercise	Week 7	Week 8
14. Rotational medicine ball throw, kneeling	3×12 reps<br>45s<br>60s	3×14 reps<br>45s<br>60s
15. Ant. oblique sling chest press with TheraBand (kneeling)	3×10 reps<br>45s<br>60s	3×12 reps<br>45s<br>60s
